# Comparative Effects of Stimulant and Antipsychotic Medications on Eating Behaviors and Weight in Children with Attention Deficit Hyperactivity Disorder

**DOI:** 10.3390/children11101189

**Published:** 2024-09-28

**Authors:** Hasan Cem Aykutlu, Esra Okyar, Mehmet Karadağ, Masum Öztürk

**Affiliations:** 1 Child and Adolescent Psychiatry Department, Trakya University Faculty of Medicine, 22030 Edirne, Turkey; 2 Department of Child and Adolescent Psychiatry, Sakarya Training and Research Hospital, 54000 Sakarya, Turkey; esraokyar@hotmail.com; 3 Child and Adolescent Psychiatry Department, Gaziantep University Faculty of Medicine, 27000 Gaziantep, Turkey; mehmetkaradag1988@gmail.com; 4 Child and Adolescent Psychiatry Department, Dicle University Faculty of Medicine, 21200 Diyarbakır, Turkey; masumozturk@hotmail.com

**Keywords:** attention deficit disorder with hyperactivity, obesity, feeding and eating disorders, child, central nervous system stimulants, antipsychotic agents, body weight, eating behavior, cross-sectional studies, drug therapy, combination

## Abstract

Background/Objectives: Attention-Deficit/Hyperactivity Disorder (ADHD) is associated with an increased risk of obesity and disordered eating behaviors. This study compared weight status and eating behaviors among drug-naïve ADHD children, those on stimulant monotherapy, those on combined stimulant and antipsychotic treatment, and healthy controls. Methods: This cross-sectional study included 547 children aged 6–12 years from four Turkish provinces: 361 with ADHD (152 drug-naïve, 156 on stimulants, and 53 on combined therapy), and 186 healthy controls. Anthropometric measurements, psychiatric assessments, and eating behavior evaluations were conducted using standardized tools. Results: Drug-naïve ADHD children had the highest obesity rate (13.8%), while those on stimulant monotherapy had the lowest (4.5%) compared to controls. Combined treatment group obesity rates were similar to controls (7.5% vs. 8.6%). The drug-naïve and combined treatment groups showed increased food approach behavior and desire to drink, with the combined treatment group also showing increased emotional overeating. Conclusions: This study reveals a complex relationship between ADHD, its pharmacological management, and the risk of obesity. Stimulant monotherapy may mitigate the risk of obesity, while combined stimulant and antipsychotic treatment may lead to problematic eating behaviors. These findings emphasize the importance of monitoring weight status and eating behaviors in ADHD children, especially those receiving pharmacological interventions.

## 1. Introduction

Attention Deficit/Hyperactivity Disorder (ADHD) is one of the most common neurodevelopmental disorders in children and adolescents, affecting approximately 5% of the population [1]. It is characterized by persistent patterns of inattention, hyperactivity, and impulsivity that significantly impair functioning and development [2]. While the core symptoms of ADHD are well-established, recent research has highlighted its association with various comorbidities and health-related behaviors, including eating disorders and obesity [3]. A meta-analysis revealed that children and adolescents with ADHD have a 40% higher prevalence of obesity compared to their healthy peers [4]. This finding is particularly concerning given the global trend of increasing childhood obesity rates. The World Health Organization (WHO) reported a more than fourfold increase in overweight and obesity among children and adolescents aged 5–19 years between 1975 and 2016 [5].

Several studies have consistently demonstrated higher rates of overweight and obesity among individuals with ADHD compared to those without the disorder. For instance, a meta-analysis by Zhu et al. (2023) found that children with ADHD were at significantly higher risk of being overweight or obese compared to controls (OR 1.56, 95% CI 1.32–1.85) [6]. The reported prevalence of obesity among children with ADHD varies considerably between studies. This is largely due to methodological differences across these studies, such as the criteria used to define obesity and the treatments employed. A recent study reported higher obesity rates among children with ADHD compared to their peers, using both the WHO criteria (17.2% vs. 3.2%) and the International Obesity Task Force criteria (10.3% vs. 1.6%) [7].

Several factors contribute to increased obesity rates among children with ADHD. The shared neurobiological pathways between ADHD and obesity, involving the hypothalamic, executive, and reward centers of the brain, suggest a complex relationship between the two conditions [3]. The core symptoms of ADHD—inattention, hyperactivity, and impulsivity—can lead to dysregulated eating behaviors and reduced motivation for physical activity [8]. Children with ADHD may struggle to maintain focus during meals, potentially resulting in overeating, or act impulsively around food, leading to poor nutritional choices and excessive snacking [4]. The reward deficiency associated with ADHD may drive some children to seek highly palatable, calorie-dense foods as a form of self-medication. Additionally, anxiety and depression, common comorbidities of ADHD, may contribute to emotional eating as a coping mechanism [9].

These neurobiological and behavioral factors lead to various impaired eating behaviors in children with ADHD. Community-based studies have shown a significant correlation between ADHD symptoms and disordered eating patterns, such as picky eating, prolonged meal times, emotional eating, and binge eating [10,11,12]. Research indicates that over 50% of children with ADHD exhibit such behaviors, with more than 40% engaging in binge eating at least once weekly [10,13]. The impact of these impaired eating behaviors extends beyond childhood; approximately 20% of children with ADHD may develop clinically significant eating disorders, including anorexia nervosa, bulimia nervosa, and binge eating disorder, during adolescence [14].

The association between ADHD and obesity is further characterized by several abnormal eating patterns that contribute to a positive energy balance [3]. These patterns include binge eating, bulimic behaviors, skipping breakfast, evening and night eating, consumption of high-calorie foods, and emotional eating. A recent systematic review concluded that both childhood and adult ADHD are associated with an increased risk of addictive-like eating behavior, emotional overeating, binge eating, strong desire for food, food responsiveness, and food preoccupation. Furthermore, childhood ADHD is hypothesized to be associated with the development of disordered eating in late childhood or adolescence [15,16].

Stimulant medications, such as methylphenidate, are the first-line pharmacological treatment for ADHD. While these drugs effectively manage core ADHD symptoms, they are associated with several side effects, including decreased appetite, weight loss, and abdominal pain [17]. The dual impact of stimulants—reducing ADHD symptomatology while negatively affecting appetite and weight—has prompted investigations into their effects on overweight and obesity, conditions commonly observed in individuals with ADHD. A meta-analysis by AlAhmari and Uddin (2022) found that the prevalence of obesity was significantly lower in medicated ADHD patients compared to untreated patients (OR 0.65, 95% CI 0.50–0.84) [18]. Furthermore, studies have demonstrated that psychostimulant use in ADHD can ameliorate eating disorder symptoms [15].

While stimulant medications have been associated with reduced weight gain and body mass index (BMI), the treatment landscape for ADHD is evolving with the increasing use of antipsychotics. Although antipsychotics are primarily approved by the Food and Drug Administration (FDA) for managing behavioral problems in various psychiatric disorders, such as bipolar disorder and autism spectrum disorder, they are increasingly being prescribed off-label in combination with stimulants to control behavioral problems in children and adolescents with ADHD. Kamble et al. (2015) reported that 19.2% of children and adolescents on long-acting stimulants were concurrently prescribed second-generation antipsychotics for at least fourteen days [19]. Similarly, Olfson et al. (2012) found that stimulants and antipsychotics were co-prescribed in 30–54% of clinical examinations involving children and adolescents [20]. The addition of antipsychotics to the treatment regimen may counteract the weight-reducing effects of stimulants due to their metabolic side effects, such as increased appetite, weight gain, and elevated risk of diabetes [17]. However, the impact of combined stimulant and antipsychotic treatment on weight status in children with ADHD remains largely unexplored in the literature.

To date, most research examining the relationship between ADHD, weight status, and eating behaviors has primarily focused on comparing individuals with ADHD to neurotypical controls or investigating the effects of monotherapy. However, several important gaps remain in our understanding. First, the impact of combined stimulant and antipsychotic treatment on weight status and eating behaviors in children with ADHD is not well understood, despite the increasing use of this combination in clinical practice. Second, few studies have directly compared drug-naïve ADHD children with those receiving different medication regimens and healthy controls within the same study design. Third, the differential effects of various ADHD treatment approaches on eating behaviors, particularly in relation to obesity risk, are not well established.

Our study aims to address this gap in the literature by comparing eating behaviors and weight status across four distinct cohorts: (1) drug-naïve children with ADHD; (2) children with ADHD receiving stimulant monotherapy; (3) children with ADHD receiving combined stimulant and antipsychotic medication; and (4) healthy controls. This comprehensive approach allows us to elucidate the baseline differences in eating behaviors and weight status between children with ADHD and their healthy peers, assess the impact of stimulant medication on these parameters, investigate the potentially opposing effects of combined stimulant and antipsychotic treatment on weight and eating behaviors, and provide insights into the complex interplay between ADHD, its pharmacological management, and risk of obesity.

## 2. Material and Methods

### 2.1. Participants

A convenience sample of children admitted to child psychiatry and pediatric clinics was recruited in a cross-sectional, multi-regional study across four Turkish provinces: Edirne (northwest), Sakarya (central), Mardin (east), and Gaziantep (south).

The study group comprised patients aged 6 to 12 years who were admitted to child psychiatry clinics and diagnosed with ADHD. Inclusion criteria were the absence of chronic medical illnesses and the absence of psychiatric diagnoses other than Oppositional Defiant Disorder (ODD), Conduct Disorder (CD), and Specific Learning Disorder. The participants were divided into three subgroups:

1. Drug-Naïve Group (DNG): this group included children diagnosed with ADHD for the first time and with no history of psychotropic treatment.
2. Stimulant Treatment Group (STG): this subgroup consisted of patients who had been using methylphenidate for at least 2 months and had a consistent history of regular daily medication use over the past month.
3. Stimulant and Antipsychotic Treatment Group (SATG): participants in this group had been taking both methylphenidate and antipsychotics (either aripiprazole or risperidone) for a minimum of 2 months and had a consistent history of regular daily medication use over the past month.

The control group included healthy children aged 6 to 12 years who were admitted to pediatric clinics. These children had no history of chronic medical illness or medication use and were not diagnosed with any psychiatric disorder based on the Kiddie Schedule for Affective Disorders and Schizophrenia for School-Age Children-Present and Lifetime DSM-5 Version (K-SADS-PL-DSM-5) assessment.

### 2.2. Measures

The K-SADS-PL-DSM-5 was administered as a semi-structured interview to assess psychopathology in both the ADHD and control groups. Interviews were conducted with children and their caregivers. Sociodemographic data were obtained using a standardized form. To assess the severity of ADHD in children, caregivers completed the Conners Parent Rating Scale-Revised Short Form (CPRS-RS). Additionally, caregivers completed the Child Eating Behavior Questionnaire (CEBQ).

The weight of children in both the research and control groups was measured in kilograms using a digital scale. Children were weighed without shoes and outer clothing. Height was measured for each child using the same scale’s flat indicator, accurate to 1 cm. Age- and sex-specific height, weight, and BMI z-scores were calculated using reference values established for Turkish children [21] via an online calculation tool [22]. Underweight (BMI-z ≤ −2), normal weight (−2 < BMI-z < 1), overweight (1 ≤ BMI-z < 2), and obese (BMI-z ≥ 2) were defined according to WHO Child Growth Standards [23].

### 2.3. The Kiddie Schedule for Affective Disorders and Schizophrenia for School-Age Children-Present and Lifetime DSM-5

The K-SADS-PL-DSM-5 is a semi-structured diagnostic interview designed to assess current and past episodes of psychopathology in children and adolescents according to DSM-5 criteria [24]. It is administered by interviewing the parent(s) and the child, and finally achieving summary ratings which include all sources of information. The K-SADS-PL-DSM-5 covers over 20 psychiatric disorders. The Turkish adaptation of the K-SADS-PL-DSM-5 demonstrated good to excellent reliability and validity across multiple psychiatric disorders [25].

### 2.4. The Conners’s Parent Rating Scale-Revised Short Form

The CPRS-RS is a questionnaire used in the diagnosis and follow-up processes of ADHD that is administered to parents [26]. It utilizes a 4-point Likert scale, with score values ranging from 0–3 for each item. Higher scores indicate that the child exhibits more of the ADHD-related problems described in the questionnaire. The CPRS-RS consists of 27 items across four subscales: Oppositional, Cognitive Problems/Inattention, Hyperactivity, and ADHD Index. Validity and reliability studies of the CPRS-RS have been conducted for the Turkish population [27].

### 2.5. The Children’s Eating Behavior Questionnaire

The CEBQ is a parent-report measure used to assess eating styles in children [28]. It consists of 35 items rated on a 5-point Likert scale ranging from 1 (never) to 5 (always). The CEBQ measures eight dimensions of eating behavior: Food Responsiveness (FR), Enjoyment of Food (EF), Emotional Overeating (EO), Desire to Drink (DD), Satiety Responsiveness (SR), Slowness in Eating (SE), Emotional Undereating (EU), and Food Fussiness (FF). Higher scores on each subscale indicate a greater presence of that eating behavior. The initial four subscales (FR, EF, DD, and EO) are identified as “food-approach” subscales, indicating positive tendencies towards eating. In contrast, the remaining four subscales (SR, SE, EU, and FF) are categorized as “food-avoidant” subscales, reflecting negative tendencies towards food consumption. In our study, we used the results from these subscales to classify subjects as either food-approaching or food-avoidant, following the methodology established during the validation of the CEBQ [29]. The validity and reliability of the CEBQ have been established in multiple countries, including a Turkish validation study demonstrating good psychometric properties for use with Turkish children [30].

### 2.6. Data Analysis

The statistical analysis was conducted using IBM SPSS Statistics for Windows, version 19.0 (IBM Corp., Armonk, NY, USA). Descriptive statistics were used to summarize the quantitative variables, including mean, standard deviation, median, minimum, and maximum values, as well as frequencies and ratios for categorical data. The Kolmogorov–Smirnov test was employed to assess the distribution of the variables. For quantitative data, the independent *t*-test was used when parametric test assumptions were met; otherwise, the Mann–Whitney U test was applied. Qualitative data were analyzed using the chi-square test, with Fisher’s exact test utilized when chi-square test assumptions were not satisfied. For multiple comparisons, the Bonferroni correction was applied to adjust the *p*-values and control for Type I error. A statistical significance level of α = 0.05 was used for all analyses, with adjusted *p*-values for multiple comparisons based on the Bonferroni method.

## 3. Results

A total of 547 children were included in our study, comprising 361 with ADHD diagnosis (152 drug-naïve, 156 on stimulants, and 53 on stimulants and antipsychotics) as well as 186 healthy controls. A post-hoc power analysis was conducted based on the FR values between groups. With an effect size of 0.144, a 5% margin of error, and our total sample size of 547 participants, the study’s power was calculated to be 81.8%.

The mean age of our sample was 8.5 ± 1.7 years, and 73.7% were male. Significant age differences were found between groups (*p* < 0.001). DNG and CG were similar in age but younger than ADHD children on medication (DNG = CG < STG = SATG, Table 1). This difference is attributed to the time required for initiating drug treatment and reaching effective dosage. However, no significant gender differences were observed between groups.

Comparing demographics, parental education levels did not differ significantly between groups. However, CG had a higher proportion of families with middle- and high-income levels compared to other groups (*p* < 0.001, Table 1).

### 3.1. Medication Patterns, Psychiatric Comorbidities, and Symptom Severity across ADHD Groups

Children receiving medication had been using their current treatment for a median of 4 months (3 months for the STG; 5 months for the SATG), and had been using stimulant treatment for 12 months (12 months for the STG; 15 months for the SATG). The majority of cases used long-acting methylphenidate, with median doses of 20 mg/day for the STG and 27 mg/day for the SATG. Among antipsychotic users, 84.9% were taking an average of 1 mg/day risperidone, while 15.1% were taking an average of 4 mg/day aripiprazole (Table 2).

Comorbid psychiatric disorders were identified in 41% of the ADHD group. These were, in order of prevalence, specific learning disorder (20.8%), ODD (19.9%), and CD (4.4%). As expected, these comorbidities were more frequent in the SATG compared to the STG and DNG (66% vs. 32.1% vs. 41.4%; *p* < 0.001). The comorbidities contributing to this difference were identified as ODD and CD (Table 2).

DNG had higher CPRS scores compared to other groups, while the control group had the lowest scores across all CPRS subscales (*p* < 0.001). The DNG and the SATG had equivalent scores on the CPRS total and oppositional scales, which were higher than the STG. On the hyperactivity and cognitive subscales, the DNG scored highest, while the STG and SATG had similar scores. No differentiation was found among ADHD groups on the hyperactivity subscale (Table 2).

### 3.2. Anthropometric Measures and Weight Status

While differences in height, weight, and BMI scores were observed between groups, these differences disappeared when evaluated using age- and sex-adjusted z-scores (Table 3).

When cases were classified according to BMI z-scores, similar distributions were observed across groups for normal weight and overweight categories. However, the frequency of obesity was significantly higher in the DNG, while it was significantly lower in the STG (*p* = 0.010). The obesity frequency was similar in the SATG and the CG. The CG showed a significantly higher frequency of underweight children, while the DNG had a significantly lower frequency. The prevalence of underweight children was similar between the STG and the SATG (Table 4).

### 3.3. Eating Behaviors

The CEBQ FA total score did not differ significantly between groups, while the food approach total score showed significant differences (*p* = 0.002). The DNG and the SATG had the highest scores among all groups. The CG and STG had similar scores, which were the lowest among all groups. A similar pattern was observed in the DD score (*p* < 0.001). On the EO subscale, the SATG had the highest score among all groups, while the CG, DNG, and STG had similar scores (*p* = 0.005). Other CEBQ subscale scores did not differ significantly between groups (Table 5).

## 4. Discussion

Our study compared weight status and eating behaviors across four groups of children: those diagnosed with ADHD who were medication naïve, those receiving only stimulant treatment, those receiving both stimulant and antipsychotic treatment, and healthy controls. Height, weight, and BMI z-scores, were found to be similar across all groups. However, when categorizing weight status according to BMI z-scores, we observed a higher prevalence of obesity in the DNG and a lower prevalence in the STG. Regarding eating behaviors associated with appetite, both the DNG and the SATG demonstrated increased eagerness to eat and passion for drinking. Additionally, the SATG exhibited increased emotional overeating behavior. These obesity-related outcomes suggest that ADHD and its treatments significantly influence weight status and eating behaviors in children.

Obesity is a well-documented comorbidity in ADHD [4,6]. Studies focusing on medication-naïve children with ADHD support this relationship, although reported rates of obesity (13–23%) and overweight (20.8–22.5%) vary across studies [31,32]. Our findings align with this trend, revealing obesity and overweight rates of 13.8% in the DNG—the highest among all groups studied. Notably, we observed fewer underweight children in ADHD groups compared to the control group. While the variability in reported rates may be attributed to factors such as weight status cut-off values, sample collection sites, comorbidities affecting weight, and medication use [7], our results underscore an elevated obesity risk, particularly in medication-naïve children with ADHD.

The weight status of children with ADHD receiving pharmacological treatment differs markedly from their non-medicated counterparts. A meta-analysis by Cortese et al. (2016) revealed a reduced obesity risk in individuals receiving ADHD medication. This finding is corroborated by studies reporting significant BMI reductions in overweight and obese children treated with methylphenidate [33], and decreased BMI z-scores following 18 months of psychostimulant use [34]. Gürbüz et al. (2016) observed significant decreases in body weight z-score, BMI, and BMI z-score, alongside increased leptin and decreased ghrelin levels, in children and adolescents with ADHD after 3 months of extended-release OROS methylphenidate treatment. However, a few studies with limited sample sizes reported no effect of ADHD treatment on weight status in children [7,35]. Our study aligns with the majority of findings, revealing the lowest obesity frequency in the STG, supporting the notion that stimulant use may decrease obesity in ADHD. Nevertheless, the long-term efficacy of stimulants in weight management for ADHD patients remains questionable. Longitudinal studies suggest that the weight-reducing effect of stimulants is transient, typically dissipating after 2–3 years [36,37]. This temporal limitation indicates that while stimulant use may offer short-term benefits, it may be insufficient for long-term weight management in ADHD patients.

Research on the weight status of children with ADHD using both stimulants and antipsychotics is limited, with existing studies presenting conflicting results. Geuijen et al. (2019) found a higher prevalence of overweight in children with ADHD compared to the population average, but found no significant association between psychostimulant, antipsychotic, or melatonin use and overweight status [38]. In contrast, Mellström et al. (2020) reported that daily methylphenidate dose was associated with decreased BMI scores, while concomitant psychotropic drug use (e.g., antipsychotics and antidepressants) correlated with increased BMI scores [33]. Our study revealed comparable obesity prevalence in the SATG and the control group, but higher rates than the STG. These findings suggest that despite the known appetite-stimulating and weight-gaining side effects of antipsychotics, their use in combination with stimulants may not significantly increase obesity risk in children with ADHD. This could potentially be attributed to the appetite-reducing effects of stimulants counterbalancing the weight-gain propensity of antipsychotics.

Our findings revealed that the DNG exhibited the highest scores in food approach behaviors and DD, aligning with previous research on atypical eating behaviors in ADHD. Wu et al. (2023) reported increased DD and FR in children with ADHD, particularly those not using medication. Their study also revealed positive correlations between fat mass percentage and inattention symptoms, as well as between DD scores and hyperactivity/impulsivity symptoms [32]. Supporting these findings, Aykutlu and Görker (2019) found elevated DD scores exclusively in drug-naïve ADHD patients [31]. The heightened DD findings are particularly noteworthy given the established link between ADHD and increased consumption of sweetened beverages [31,39]. This association is concerning because sweetened beverage consumption has a strong connection to fat mass gain [40]. Furthermore, longitudinal studies have consistently demonstrated associations between ADHD symptoms and various eating behaviors, including emotional overeating, FR, and desire for food across different age groups from childhood to adolescence [16,41]. These persistent atypical eating patterns may contribute to the increased prevalence of obesity observed among individuals with ADHD, highlighting the importance of addressing eating behaviors in ADHD management strategies.

The similarity in eating behaviors between the STG and the CG in our study is noteworthy, as it supports the notion that stimulant medications may help normalize eating patterns in ADHD. This finding aligns with previous research demonstrating that stimulant treatment can improve impaired eating behaviors and provide weight control in individuals with ADHD [34]. However, Bowling et al.’s (2017) longitudinal study found that both medicated and non-medicated children with ADHD maintained higher unhealthy diet scores compared to controls [37]. Similarly, in another longitidunal study, no difference was found in disordered eating behaviors between stimulant-treated and untreated adolescents with ADHD [42]. It is worth noting that both studies relied on parental or self-reports of ADHD diagnosis and treatment, which could potentially confound the findings. While the exact mechanism remains unclear, it is hypothesized that stimulants improve executive functioning and reduce impulsivity, potentially benefiting children’s eating behavior regulation [4]. Our findings suggest that increasing medication compliance in children with ADHD could potentially serve as a protective factor against obesity risk, though further research is needed to establish a causal relationship.

Our study revealed that the SATG demonstrated the highest total food approach score and DD, comparable to the DNG. Notably, the SATG group also exhibited the highest EE score. To our knowledge, this is the first study to specifically examine eating behaviors in children with ADHD receiving both stimulant and antipsychotic medications. These findings align with and extend previous research in several ways. El Archi et al. (2020) posited that negative affectivity and emotion dysregulation induce addictive-like eating behaviors in ADHD [15]. Additionally, established correlations exist between severe impulsivity and binge/bulimic eating behaviors, as well as between symptom severity and disordered eating behaviors [43,44]. Our results may therefore reflect persistent ADHD and oppositional symptoms that continue despite ongoing treatment, particularly given that the SATG clinical group has been characterized by higher ADHD symptom severity and higher rates of ODD and CD comorbidity compared to the stimulant-only group [45]. However, the interpreting of these results is complicated by the concurrent use of stimulants and antipsychotics, which exert opposing effects on appetite regulation. Stimulants typically suppress appetite, while antipsychotics often induce weight gain through increased appetite. This pharmacological dichotomy introduces significant complexity when assessing eating behaviors in children with ADHD under combination therapy. The intricate interplay between ADHD symptomatology, medication effects, and eating patterns underscores the necessity for longitudinal research. Such studies are imperative to disentangle the multifaceted factors influencing appetite and weight trajectories in this population. Specifically, long-term investigations should aim to elucidate how combined stimulant and antipsychotic use affects eating behaviors over time, accounting for developmental changes, medication dosage adjustments, and potential compensatory mechanisms.

Our study has limitations that warrant consideration. Foremost is the relatively small sample size in the SATG compared to the other groups. This limitation stems from the infrequent off-label use of antipsychotics in ADHD treatment, presenting challenges in participant recruitment. The small sample size may reduce the statistical power of our analyses, potentially limiting our ability to detect subtle differences between groups or generalize findings to the broader population of children with ADHD treated with stimulants and antipsychotics.

Additionally, several potential confounding factors may have influenced our findings regarding weight and eating behaviors independent of ADHD medication. Consistent with previous research [46], we observed that children with ADHD often come from families with lower socioeconomic status compared to controls, potentially impacting our results. Lower socioeconomic status limits access to nutritious foods and safe environments for physical activity, contributing to higher obesity rates in children [47]. Furthermore, dietary patterns may act as a confounding factor, as research has shown that children with ADHD tend to consume higher proportions of energy-dense, nutrient-poor foods and sugar-sweetened beverages, which are consistently associated with increased weight gain and obesity risk [31,39,40]. Beyond dietary factors, physical activity levels are also crucial; studies have shown that children with ADHD may engage in lower levels of physical activity compared to their peers, further contributing to higher BMIs [48]. Moreover, genetic predisposition plays a role, as a family history of obesity can predispose children with ADHD to weight gain due to both genetic and shared environmental factors [38]. These interconnected factors of socioeconomic status, diet, physical activity, and genetic predisposition may confound the relationship between ADHD medication and weight gain, emphasizing the importance of considering them in future research.

It is also important to note that our findings are specific to the age group studied (6–12 years old) and may not be generalizable to other age groups. Furthermore, given the underrepresentation of girls in our sample, results should be interpreted with caution when referring to girls with ADHD. This limitation in demographic representation underscores the need for more diverse and inclusive samples in future studies.

Lastly, the cross-sectional design of our study limits causal inferences. Longitudinal studies would be beneficial to elucidate the temporal relationships between ADHD, medication use, and changes in weight and eating behaviors over time. Such approaches would allow for a more nuanced understanding of the relative contributions of ADHD symptoms, medication effects, and other environmental and genetic factors to weight and eating behaviors in this population.

Despite these limitations, our study possesses several strengths. A key advantage lies in our large clinical sample and rigorous exclusion criteria, which eliminated medical and psychiatric comorbidities known to influence weight and eating behaviors. This is particularly noteworthy given that common psychiatric comorbidities in ADHD, such as depression and anxiety disorders, have been consistently associated with increased rates of overweight and obesity in this population [12,49,50]. Furthermore, our multicenter approach, drawing participants from diverse sociocultural regions, contributes to the generalizability of our results. Complementing these design strengths, our study’s methodological rigor is evident in our use of standardized measurements for weight and height, and validated questionnaires for assessing eating behaviors. This approach ensures the reliability and validity of our data collection methods, allowing for more accurate comparisons across groups. Finally, the long-term, consistent treatment regimens of the ADHD participants provide valuable insights into the effects of prolonged treatment on weight and height trajectories.

## 5. Conclusions

Our study reveals that children with ADHD have a significantly higher risk of obesity and obesity-related eating behaviors compared to their typically developing peers. This finding has important clinical implications, particularly for drug-naïve children with ADHD. The high obesity rates observed in the DNG underscore the critical need for early intervention strategies targeting weight management in newly diagnosed ADHD patients. Clinicians should consider implementing nutritional counseling and lifestyle interventions as part of the initial treatment plan for ADHD, even before pharmacological interventions are initiated.

Furthermore, our results demonstrate that stimulant treatment appears to mitigate obesity risks effectively. Children in the STG showed lower obesity rates compared to the DNG, suggesting that stimulant medications may have a protective effect against weight gain. This finding supports the importance of medication adherence in ADHD management, not only for symptom control but also for potential weight regulation benefits.

Notably, while children with severe ADHD and high impulsivity receiving combined stimulant and antipsychotic treatment did not show a significantly increased risk of obesity, they did manifest a higher prevalence of obesity-related eating behaviors. This complex interplay between medications highlights the need for careful monitoring of eating patterns in patients receiving combination therapy. Clinicians should be aware of this potential interaction and consider implementing targeted dietary interventions and regular weight monitoring for patients on combined therapy.

Given these findings, we recommend that clinicians thoroughly evaluate the eating behaviors and dietary patterns of children with ADHD, in addition to assessing ADHD symptomatology. This comprehensive approach should be applied across all treatment stages, from initial diagnosis through various pharmacological interventions. Moreover, ensuring consistent adherence to prescribed ADHD treatments appears to be beneficial in preventing obesity in this high-risk population.

## Figures and Tables

**Table 1:** Demographic and clinical characteristics of the study group.

Variables		ADHD Group (n = 361)			CG (n = 186)	*p*/Post-Hoc
		DNG (n = 152)	STG (n = 156)	SATG (n = 53)		
Age		7.91 (2.16)	9.37 (2.5)	9.00 (2.63)	7.67 (2.41)	**<0.001 ^MU^** DNG = CG < STG = SATG
Sex	Boy	75% (n = 114)	74.4% (n = 116)	83% (n = 44)	69.4% (n = 129)	0.226 ^CS^
	Girl	25% (n = 38)	25.6% (n = 40)	17% (n = 9)	30.6% (n = 57)	
Family income	Low	52% (n = 79)	39.4% (n = 61)	56.6% (n = 30)	26.3% (n = 49)	**<0.001 ^CS^** CG > SATG > DNG = STG (high income)
	Medium	42.8% (n = 65)	54.8% (n = 85)	35.8% (n = 19)	53.8% (n = 100)	
	High	5.3% (n = 8)	5.8% (n = 9)	7.5% (n = 4)	19.9% (n = 37)	
Mother education	Low	30.9% (n = 47)	31.2% (n = 48)	24.5% (n = 13)	36% (n = 67)	0.583 ^CS^
	Medium	46.1% (n = 70)	45.5% (n = 70)	47.2% (n = 25)	37.6% (n = 70)	
	High	23% (n = 35)	23.4% (n = 36)	28.3% (n = 15)	26.3% (n = 49)	
Father education	Low	22.4% (n = 34)	20.6% (n = 32)	22.6% (n = 12)	22.7% (n = 42)	0.746 ^CS^
	Medium	53.3% (n = 81)	55.5% (n = 86)	56.6% (n = 30)	47.6% (n = 88)	
	High	24.3% (n = 37)	23.9% (n = 37)	20.8% (n = 11)	29.7% (n = 55)	

Abbreviations: ADHD: Attention Deficit/Hyperactivity Disorder, DNG: Drug-Naïve Group, STG: Stimulant Treatment Group, SATG: Stimulant and Antipsychotic Treatment Group, CG: Control group. ^CS^: Chi-Square Test, ^MU^: Mann–Whitney U Test. Median (interquartile range) or % (n).

**Table 2:** Clinical characteristics of the study sample.

Variables		ADHD Group (n = 361)			CG (n = 186)	*p*/Post-Hoc
		DNG (n = 152)	STG (n = 156)	SATG (n = 53)		
Psychiatric comorbidity		41.4% (63)	32.1% (50)	66% (35)	-	**<0.001 ^CS^** SATG > DNG > STG
	SLD	20.4% (31)	25% (39)	9.4% (5)	-	0.054 ^CS^
	ODD	21.1% (32)	8.3% (13)	50.9% (27)	-	**<0.001 ^CS^** SATG > DNG > STG
	CD	3.9% (6)	2.6% (4)	11.3% (6)	-	**0.026 ^CS^** SATG > DNG = STG
CRS-Oppositional		10 (10)	7 (8)	11 (7)	4 (6)	**<0.001 ^KW^** DNG = SATG > STG > CG
CRS-Cognitive		13 (6)	10 (9)	9 (9)	2 (4)	**<0.001 ^KW^** DNG > SATG = STG > CG
CRS-Hyperactivity		11 (8)	7 (9)	8 (9)	2 (4)	**<0.001 ^KW^** DNG = SATG = STG > CG
CRS-ADHD index		25.5 (11)	20 (16)	21 (14)	6 (9)	**<0.001 ^KW^** DNG > SATG = STG > CG
CRS total score		53.5 (25)	40 (33)	45 (31)	13 (18)	**<0.001 ^KW^** DNG > SATG = STG > CG
Duration of current treatment at regular use (month)		n/a	3 (1–60)	5 (1–72)	n/a	
Duration of stimulant treatment (month)		n/a	12 (1–60)	15 (1–72)	n/a	
Methylphenidate extended-release		n/a	89.1% (n = 139)	90.6% (n = 48)	n/a	
Dose (mg/day)		n/a	20 (10–54)	27 (10–54)	n/a	
Methylphenidate short acting		n/a	17.3% (n = 27)	15.1% (n = 8)	n/a	
Dose (mg/day)		n/a	10 (5–30)	10 (10–25)	n/a	
Risperidone		n/a	n/a	84.9% (n = 45)	n/a	
Dose (mg/day)		n/a	n/a	1 (0.25–1.5)	n/a	
Aripiprazole		n/a	n/a	15.1% (n = 8)	n/a	
Dose (mg/day)		n/a	n/a	4 (1–10)	n/a	

Abbreviations: ADHD: Attention Deficit/Hyperactivity Disorder, DNG: Drug Naïve Group, STG: Stimulant Treatment Group, SATG: Stimulant and Antipsychotic Treatment Group, CG: Control group, CRS: Conners Rating Scale, SLD: Specific Learning Disorder, ODD: Oppositional Defiant Disorder; CD: Conduct Disorder. ^CS^: Chi-Square Test, ^KW^: Kruskal–Wallis test. Median (interquartile range/min-max) or % (n). n/a: Not applicable.

**Table 3:** Comparison of the growth measures between ADHD and control groups.

Variables	ADHD Group (n = 361)			Control Group (n = 186)	*p*
	DNG (n = 152)	STG (n = 156)	SATG (n = 53)		
Height	130 (13.88)	137 (16.38)	135 (16.25)	130 (14.88)	**<0.001** **^KW^**
Height z score	0.53 ± 1.22	0.52 ± 1.09	0.67 ± 0.90	0.60 ± 1.28	0.777 ^OA^
Weight	28.35 (11)	32.00 (13)	32.70 (11.65)	26.00 (12)	**<0.001** **^KW^**
Weight z score	0.60 ± 1.33	0.44 ± 1.10	0.58 ± 1.16	0.40 ± 1.19	0.402 ^OA^
BMI	16.88 (4.3)	16.97 (3.86)	17.07 (4.19)	16.22 (4.01)	**0.035** **^KW^**
BMI z score	0.34 ± 1.32	0.14 ± 1.17	0.18 ± 1.41	0.04 ± 1.35	0.219 ^OA^

Abbreviations: ADHD: Attention Deficit/Hyperactivity Disorder, DNG: Drug-Naïve Group, STG: Stimulant Treatment Group, SATG: Stimulant and Antipsychotic Treatment Group, CG: Control group, BMI: Body Mass Index; ^KW^: Kruskal–Wallis test, ^OA^: One-way ANOVA test. Median (interquartile range) or Mean (standard deviation).

**Table 4:** Comparison of BMI groups between ADHD and control groups.

Variables		ADHD Group (n = 361)			CG (n = 186)	*p*	Post Hoc
		DNG (n = 152)	STG (n = 156)	SATG (n = 53)			
BMI z score groups	Wasted	2% (3)	3.8% (6)	5.7% (3)	9.1% (17)	**0.010 ^CS^**	CG > STG = SATG > DNG
	Normal	70.4% (107)	70.5% (110)	62.3% (33)	66.7% (124)		Similar
	Overweight	13.8% (21)	21.2% (33)	24.5% (13)	15.6% (29)		Similar
	Obese	13.8% (21)	4.5% (7)	7.5% (4)	8.6% (16)		DNG > SATG = CG > STG

Abbreviations: ADHD: Attention Deficit/Hyperactivity Disorder, DNG: Drug-Naïve Group, STG: Stimulant Treatment Group, SATG: Stimulant and Antipsychotic Treatment Group, CG: Control group, BMI: Body Mass Index; ^CS^: Chi-Square Test, % (n).

**Table 5:** Eating behaviors across groups.

Variable	ADHD Group (n = 361)			CG (n = 186)	*p*/Post-Hoc
	DNG (n = 152)	STG (n = 156)	SATG (n = 53)		
**Food responsiveness**	10 (7)	9 (8)	10 (9)	9 (5)	0.113 ^KW^
**Emotional overeating**	6 (6)	6 (6)	8 (7)	5.5 (4)	**0.005 ^KW^** SATG > CG = STG = DNG
**Enjoyment of food**	16.5 (10)	16 (9)	16 (7)	15 (7)	0.327 ^KW^
**Desire to drink**	9 (6)	8 (6)	10 (4)	8 (6)	**<0.001 ^KW^** SATG = DNG > CG = STG
**Food approach**	41 (19)	40 (21)	44 (16)	36 (17)	**0.002 ^KW^** SATG = DNG > CG = SG
**Satiety responsiveness**	20.5 (10)	21 (7)	23 (9)	21 (10)	0.139 ^KW^
**Slowness in eating**	8 (6)	8 (7)	8 (6)	9 (6)	0.331 ^KW^
**Emotional undereating**	10 (6)	10 (4)	11 (3)	10 (5)	0.573 ^KW^
**Food fussiness**	7 (4)	8 (4)	7 (5)	7 (5)	0.655 ^KW^
**Food avoidance**	47.77 ± 10.98	48.54 ± 10.89	49.13 ± 9.85	48.67 ± 10.43	0.821 ^OA^

Abbreviations: ADHD: Attention Deficit/Hyperactivity Disorder, DNG: Drug-Naïve Group, STG: Stimulant Treatment Group, SATG: Stimulant and Antipsychotic Treatment Group, CG: Control group. ^KW^: Kruskal–Wallis test, ^OA^: One-way ANOVA test. Median (interquartile range) or mean (standart deviation).

## Data Availability

The raw data supporting the conclusions of this article will be made available by the authors on request from the corresponding author due to privacy reasons.
